# The sodium/ascorbic acid co-transporter SVCT2 distributes in a striated membrane-enriched domain at the M-band level in slow-twitch skeletal muscle fibers

**DOI:** 10.1186/s40659-024-00554-6

**Published:** 2024-11-06

**Authors:** Daniel Sandoval, Jessica Mella, Jorge Ojeda, Francisca Bermedo-García, Marcela Low, Sylvain Marcellini, Maite A. Castro, Mariana Casas, Enrique Jaimovich, Juan Pablo Henríquez

**Affiliations:** 1 Neuromuscular Studies Lab (NeSt Lab), Instituto de Anatomía, Histología y Patología, Facultad de Medicina, Universidad Austral de Chile, Valdivia, 5110566 Chile; 2 Carrera de Química y Farmacia, Facultad de Medicina y Ciencia, Universidad San Sebastián, Valdivia, 5090000 Chile; 3 Departamento de Biología Celular, Facultad de Ciencias Biológicas, Universidad de Concepción, Concepción, 4070386 Chile; 4 Instituto de Bioquímica y Microbiología, Facultad de Ciencias, Universidad Austral de Chile, Valdivia, 5110566 Chile; 5 Instituto de Ciencias Biomédicas, Facultad de Medicina, Universidad de Chile, Santiago, 8320000 Chile; 6 Facultad de Ciencias Veterinarias, Universidad de Concepción, Chillán, 3812120 Chile; 7 Facultad de Odontología y Ciencias de la Rehabilitación, Universidad San Sebastián, Concepción, Chile

**Keywords:** SVCT2, Vitamin C, Skeletal muscle, M-band, Sarcoplasmic reticulum

## Abstract

**Background:**

Vitamin C plays key roles in cellular homeostasis, functioning as a potent antioxidant and a positive regulator of cell differentiation. In skeletal muscle, the vitamin C/sodium co-transporter SVCT2 is preferentially expressed in oxidative slow fibers. SVCT2 is up-regulated during the early fusion of primary myoblasts and decreases during initial myotube growth, indicating the relevance of vitamin C uptake via SVCT2 for early skeletal muscle differentiation and fiber-type definition. However, our understanding of SVCT2 expression and function in adult skeletal muscles is still limited.

**Results:**

In this study, we demonstrate that SVCT2 exhibits an intracellular distribution in chicken slow skeletal muscles, following a highly organized striated pattern. A similar distribution was observed in human muscle samples, chicken cultured myotubes, and isolated mouse myofibers. Immunohistochemical analyses, combined with biochemical cell fractionation experiments, reveal a strong co-localization of SVCT2 with intracellular detergent-soluble membrane fractions at the central sarcomeric M-band, where it co-solubilizes with sarcoplasmic reticulum proteins. Remarkably, electrical stimulation of cultured myofibers induces the redistribution of SVCT2 into a vesicular pattern.

**Conclusions:**

Our results provide novel insights into the dynamic roles of SVCT2 in different intracellular compartments in response to functional demands.

**Supplementary Information:**

The online version contains supplementary material available at 10.1186/s40659-024-00554-6.

## Introduction

The skeletal muscle tissue continuously balances the production of reactive metabolites resulting from increasing oxygen consumption and the subsequent generation of reactive oxygen species (ROS) [1]. Oxidative stress is considered one of the most relevant triggers of the imbalance between protein synthesis and degradation that causes muscle atrophy [2–5]. Stress conditions like prolonged exercise, aging, and certain pathologies induce potentially harmful ROS [1, 6, 7]. This is led through distinct intracellular sources such as NADPH oxidases (NOX), mitochondria, the endoplasmic reticulum, peroxisomes, the Golgi apparatus, and the nucleus, which increase lipid peroxidation, protein carbonylation, and alter glutathione redox status, among others [4].

As the redox imbalance represents a permanent challenge, skeletal muscle has evolved efficient enzymatic and non-enzymatic antioxidant mechanisms [8, 9]. The reduced form of vitamin C, ascorbic acid, has been characterized as one of the main antioxidants in plasma and various tissues [10–12]. As humans are unable to synthesize vitamin C, plasma concentrations are strictly regulated by transporters, which are required for vitamin C entry into the cytosol and intracellular organelles [13, 14]. Thus, whereas the oxidized form of vitamin C, dehydroascorbic acid (DHA), can be taken up by members of the facilitative hexose transporters GLUTs [15, 16], its reduced ascorbic acid form is transported by the high affinity sodium/vitamin C co-transporters SVCT1 and SVCT2 [17–20]. Remarkably, the skeletal muscle tissue contains around 40% of the whole-body ascorbic acid [21], an essential antioxidant to maintain muscle functions [22]. Indeed, ascorbic acid deficiency leads to muscle atrophy and deterioration of physical performance [23, 24]. However, the mechanisms mediating ascorbic acid bioavailability in skeletal muscle have been poorly described.

Original findings showed that the expression of the SVCT2 transporter is inversely modulated by the presence of the antioxidant lipoate [25]. Our evidence demonstrated that SVCT2 is expressed in the skeletal muscle tissue in both developing and adult chicken [26, 27]. Consistent with the role of vitamin C on cell differentiation [28–30], we also found that the early fusion of chicken primary myoblasts into myotubes is accompanied by SVCT2 upregulation [31], while the levels of SVCT2 decrease upon postnatal muscle growth [27]. In line with its antioxidant effects, our reports showed that SVCT2 displays a restricted expression in oxidative slow-twitch muscle fibers [26, 31]. Together, these findings suggest that SVCT2-mediated uptake of vitamin C could play diverse roles on skeletal muscle development and physiology.

Our previous work has shown that SVCT2 is distributed intracellularly [26, 31]. Consistently, SVCT2 distributes intracellularly in other models including cell lines and tissues [32, 33]. For instance, while breast cancer cells and differentiating myoblasts exhibit SVCT2 distribution in the plasma membrane and the mitochondrial membrane [34, 35], SVCT2 is localized at the plasma membrane and the endoplasmic reticulum membrane in neurons [36]. Interestingly, the localization and dynamic distribution of SVCT2 between cell compartments and the plasma membrane are crucial for different cell types and contexts [37–39]. However, the intracellular localization and potential function of SVCT2 in skeletal muscle fibers has not been yet addressed.

In this work, we have conducted immunohistochemistry and protein fractionation studies to underscore the precise intracellular localization of SVCT2. Our findings show that SVCT2 distributes in a highly ordered striated pattern exhibiting co-localization with intracellular detergent-soluble membrane fractions at central M-band of sarcomeres, from where it is co-solubilized along with sarcoplasmic reticulum (SR) proteins. Remarkably, electrical stimulation of isolated muscle fibers results in the redistribution of SVCT2 in a vesicular pattern. Our novel findings reveal that SVCT2 could play dynamic roles in different intracellular compartments according to functional demands.

## Materials and methods

### Animals

Fertilized chicken eggs were incubated at 37.5 °C in a circulated air incubator. Chicken embryos were staged according to Hamburger and Hamilton (HH stages) [40]. Male BALB/c mice (5–7 wk-old) were obtained from the Faculty of Medicine Animal Facility, Universidad de Chile. Room temperature was set at 23 °C, with a 12:12 h light–dark cycle. The human sample was obtained from the lumbar slow-twitch multifidus (transversospinal) muscle [41], from a 40 years old male donor subjected to surgical intervention after traumatic spinal cord injury at Guillermo Grant Benavente Hospital (Concepción, Chile). Experiments were conducted following the guidelines outlined in the Biosafety and Bioethics Manual of the National Commission of Scientific and Technological Research (ANID, Chilean Government). The Bioethics Committees of University of Concepcion and Universidad de Chile approved all experimental procedures carried out during this study.

## Cell cultures

Myoblasts were isolated from embryonic HH38 White Leghorn chicken slow *Medial adductor* muscle tissue, as previously described [42]. The tissue was mechanically disrupted and then treated with 0.25% trypsin (HyClone, South Logan, UT, USA) for 15 min at 37 °C under mild agitation. Cells were suspended in growth medium (DMEM high glucose (Hyclone), 20% fetal bovine serum (Hyclone), 2% chicken embryo extract and antibiotics) with repeated pipetting. The cell suspension was filtered through a triple nylon cloth filter and centrifuged 1,500 x*g* for 5 min. Cells were plated at a density of 50,000 cells per cm^2^ of 0.5% gelatine-coated culture dishes in complete medium. To induce differentiation, the growth medium was replaced with differentiation medium (DMEM high glucose, 10% horse serum plus 2.5% fetal bovine serum, 2% chicken embryo extract and antibiotics) at day 2 [31].

## Immunohistochemistry

Slow-twitch muscles from adult chickens were dissected, mounted in OCT (Sakura Finetek, Torrance, CA, USA) and quickly frozen in isopentane cooled with liquid nitrogen [43]. Longitudinal cryosections (20 μm) were immunostained with primary antibodies diluted in blocking solution (1% BSA in Tris phosphate buffer) 12–15 h at 4 ˚C. We used a combination of a polyclonal goat anti SVCT2 (Santa Cruz Biotechnology) along with mouse monoclonal antibodies raised against laminin, titin, slow myosin heavy chain, desmin, slow C-protein, the ryanodine receptor (RyR) and myomesin, all of them obtained from the Developmental Studies Hybridoma Bank at The University of Iowa, IA, USA. Corresponding alexa488 and alexa546-conjugated secondary immunoglobulins (Invitrogen) were incubated for 2 h at RT and the slides were subsequently mounted with aqueous medium for fluorescence (Sigma). Images were acquired with a laser confocal Nikon Eclipse TE2000-U microscope.

## Protein fractionation

Cultured myotubes were washed and scrapped in 400 µl of PBS containing protease inhibitors (80 mM aprotinin, 1.5 mM pepstatin A, 2 mM leupeptin, 104 mM AEBSF, 4 mM Bestatin, 1.4 mM E-64) (Sigma, St. Louis, MO, USA) and homogenized with 1 mL syringe. The homogenate was centrifuged at 12,000 x*g* for 10 min at 4 °C to obtain solubilized cytosolic proteins (PBS extract). The pellet was resuspended in a 50 mM Tris HCl pH 7.4; 0.5% v/v Triton X-100 and 0.15 M NaCl buffer, re-homogenized, and re-centrifuged in the same conditions to obtain a protein fraction enriched in cell membrane proteins (Tx extract). Finally, the sediment obtained in the previous step was resuspended in a 50 mM Tris HCl pH 7.4; 0.5% v/v Triton X-100 and 0.5 M KCl buffer and subjected to similar homogenization and centrifugation steps (Tx-KCl extract).

For total protein extracts (TE), chicken *anterior* *Latissimus dorsi *(ALD) and *Medial adductor* muscles were homogenized in buffer A (25 mM Tris HCl pH 7.4; 100 mM NaCl; 1 mM EDTA; 1 mM EGTA, and 1 mM PMSF) at 4 °C and centrifuged at 1,000 x*g* for 15 min (Sorvall SS-34 rotor). The obtained supernatant (S) was filtered through sterile gauze and centrifuged at 27,000 x*g* for 30 min, recovering a first supernatant (S1) and a pellet containing heavy microsomes (HM). In the ionic strength-detergent fractionation protocol, the HM pellet was then resuspended in buffer B (MA) (25 mM Tris HCl pH 7.4; 500 mM NaCl; 1 mM EDTA; 1 mM EGTA, and 1 mM PMSF) and re-centrifuged in the same conditions to obtain a second supernatant (S2A) and a P3A pellet. Finally, P3A was resuspended in buffer C (25 mM Tris HCl pH 7.4; 500 mM NaCl; 1 mM EDTA; 1 mM EGTA; 1 mM PMSF, and 0.5% Tx-100) and centrifuged at 17,000 x*g* for 30 min to obtain the S3A and a final P4A pellet [44]. In the detergent-ionic strength fractionation protocol, the HM pellet was resuspended in buffer D (MB) (25 mM Tris HCl pH 7.4; 100 mM NaCl; 1 mM EDTA; 1 mM EGTA; 1 mM PMSF and 0.5% Tx-100) and centrifuged at 27,000 x*g* for 30 min, obtaining a supernatant (S2B) and the pellet (P3B). Finally, P3B was resuspended in buffer C and centrifuged at 17,000 x*g* for 30 min, obtaining the supernatant (S3B) and a final P4B pellet.

## PAGE-SDS and western blot

Samples (30 µg) were loaded in each lane and fractionated by 10% polyacrylamide gel electrophoresis in the presence of sodium dodecylsulfate (PAGE-SDS). Gels were stained with a Coomassie brilliant blue (Sigma) solution (3:5:2 methanol: distilled water: acetic acid) for 15 min and further destained with warm water for 2 h. For immunoblotting, 30 µg of muscle proteins were loaded in each lane, fractionated in gels, transferred to PVDF membranes, and probed against polyclonal goat anti SVCT2 and b-actin (both from Santa Cruz Biotechnology) antibodies, as well as with mouse monoclonal antibodies raised against myomesin, the Cav1.1 receptor, the Na ^+^/K^+^-ATPase, and the sarco/endoplasmic reticulum Ca^**2**+^-ATPase 2 (SERCA 2), all of them from the Developmental Studies Hybridoma Bank. A peroxidase-conjugated secondary anti-mouse antibody (1/2,000) (Jackson Immuno Research, West Grove, PA, USA) was incubated for 2 h at room temperature. Reactions were developed with enhanced chemiluminescence according to the ECL Western blotting analysis system (Perkin Elmer, Waltham, MA, USA).

### Isolation and stimulation of adult mouse skeletal muscle fibers

BALB/c mice (5–7 wk-old) were euthanized by cervical dislocation. *Flexor digitalis brevis* (FDB) muscles from both limbs were dissected and maintained in sterile 0.01 M PBS. For muscle fiber separation, the muscles were digested in type II collagenase (450–500 U/ml) for 90 min at 37 °C under agitation at 170 rpm. Subsequently, the muscles were incubated at 37 °C in DMEM containing 10% horse serum and the fibers were mechanically separated using fire-polished Pasteur pipettes. The isolated fibers were finally seeded onto 35 mm Petri dishes pretreated with Matrigel™. The isolated fibers were maintained for 18–20 h at 37 °C, 5% CO_2_ and 95% humidity. Prior to electrical stimulation, the fibers were incubated with 25 µM BTS (N-benzyl-p-toluene sulfonamide) for 5 min to inhibit muscle contraction. Electrical stimulation of the fibers was applied with platinum electrodes connected through an isolation unit to an electrostimulator (Grass Stimulator S48). Trains of 270 square pulses of 0.3 ms duration at a frequency of 20 Hz were used. The stimulated fibers were maintained in an incubator for 2, 4, and 8 h post-stimulation in DMEM containing 10% horse serum until immunohistochemistry.

## Statistical analyses

All data are presented as mean ± SEM and analyzed using GraphPad Prism 6. Differences between groups were assessed with ANOVA. A *p* value of < 0.05 indicates statistically significant differences.

## Results

**Fig. 1:**
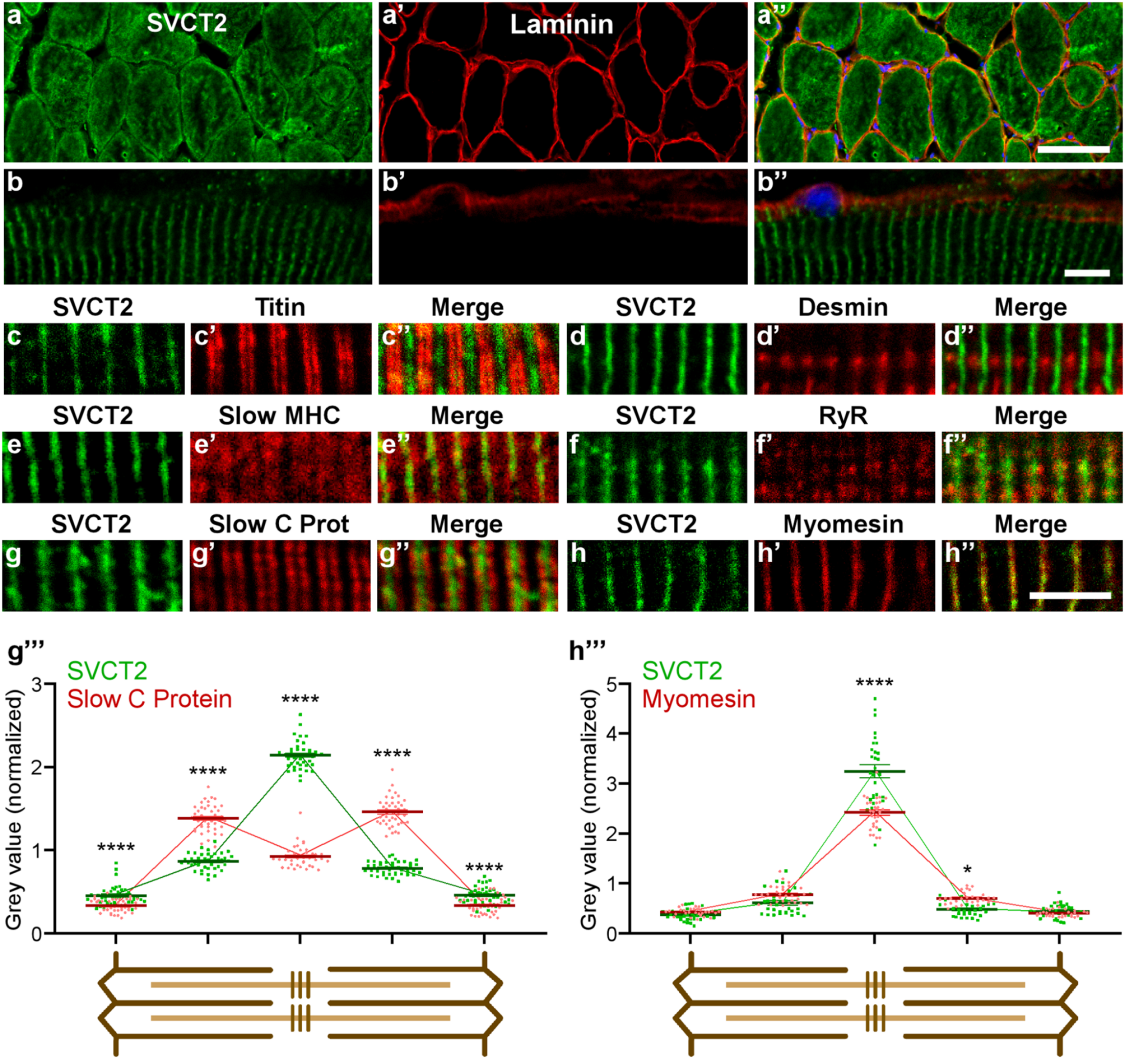
SVCT2 is distributed intracellularly in a transverse striated pattern in adult chicken muscle. Cross sections (**a-a’’**) and longitudinal sections (**b-b’’**) of 20 μm from adult chicken LDA muscles were stained using antibodies against SVCT2 (green) and laminin (red). Nuclei were counterstained with DAPI (blue). Representative images obtained by confocal microscopy show the intracellular SVCT2 distribution pattern. Scale bar = 50 μm. (**c-h’’**) Longitudinal cryosections of adult chicken LDA muscles were co-incubated with an anti-SVCT2 antibody (green) together with antibodies against titin (**c-c’’**), desmin (**d-d’’**), slow MHC (**e-e’’**), ryanodine receptor (RyR) (**f-f’’**), slow C protein (**g-g’’**), and myomesin (**h-h’’**) (in red) to investigate the intracellular distribution of SVCT2. Scale bar = 5 μm. (**g’’’** and **h’’’**) Quantification of fluorescence intensities (expressed as gray values normalized to their mean intensity) throughout the sarcomeres (schemes below plots) evidence the differential distribution between SVCT2 and slow C protein (**g’’’**), as well as the close codistribution of SVCT2 and myomesin (**h’’’**) at the M-band level. Plots represent the mean ± SEM of > 30 sarcomeres (dots) from 3 different immunohistochemical staining. * *p* < 0.05; ****p < 0.0001, Anova

**Fig. 2:**
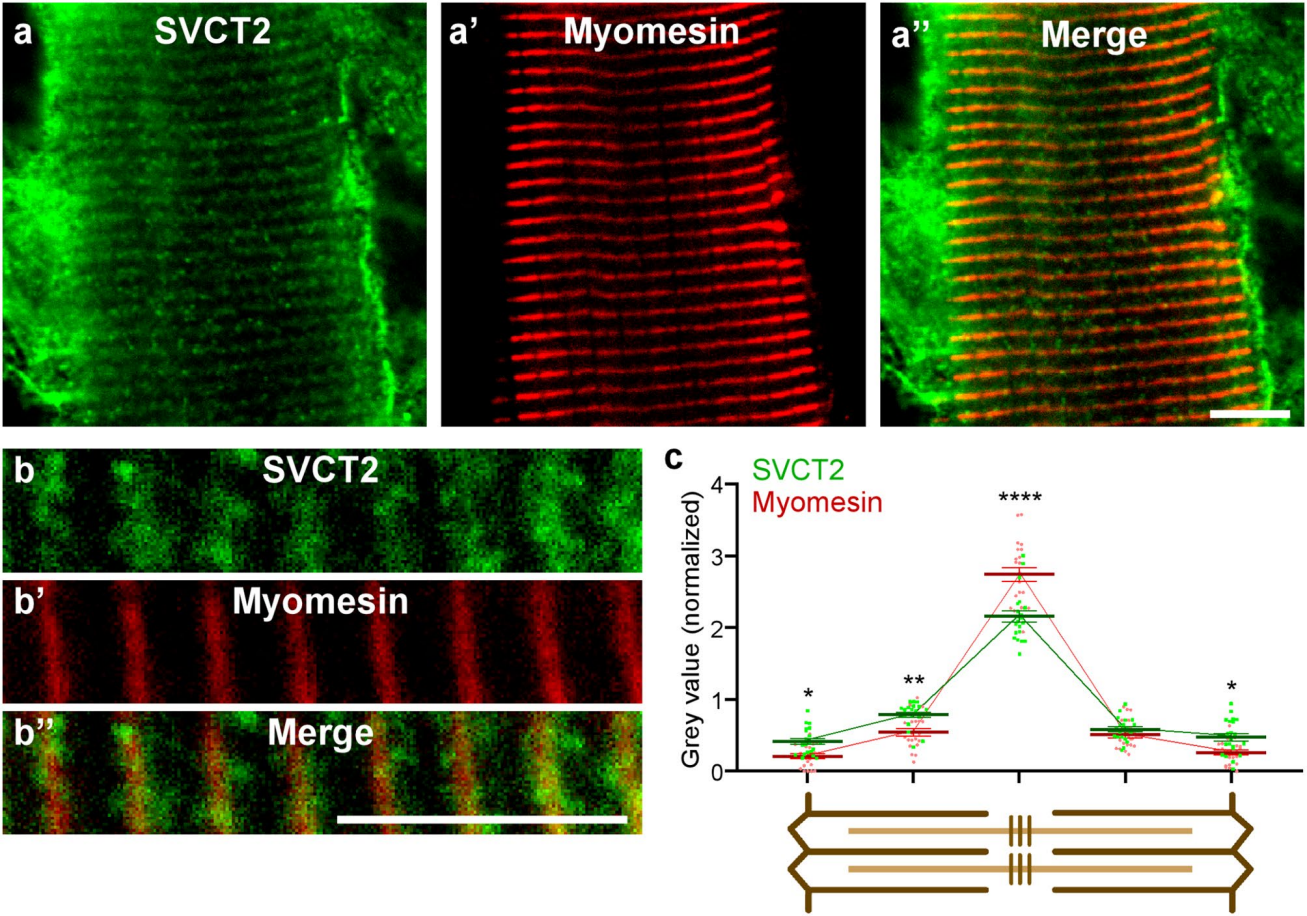
SVCT2 localizes to intracellular membrane domains in human oxidative skeletal muscle fibers. (**a-a’’**,** b-b’’**) Longitudinal cryosections of the human lumbar multifidus (transversospinal) muscle were co-incubated with primary antibodies against SVCT2 (green) and myomesin (red), as the representative confocal images are shown. Scale bar = 5 μm. (**b’’’**) Fluorescence signal for SVCT2 (green) and myomesin (red) were analyzed. Scale bar = 5 μm. **(c)** Quantification of fluorescence intensities throughout the sarcomeres (scheme below plot) evidence the close codistribution of SVCT2 and myomesin at the M-band level. Plots represent the mean ± SEM of > 15 sarcomeres (dots) from 3 different immunohistochemical staining. * *p* < 0.05; **p < 0.01; ****p < 0.0001, Anova

To first analyze the intracellular distribution of SVCT2, cryosections of the ALD muscle were co-incubated with antibodies against SVCT2 and laminin, a basal lamina protein (Figs. 1a-a’’, b-b’’). Detailed observation showed that the evident intracellular distribution of SVCT2 in transversal cryosections (Fig. 1a-a’’) corresponded to a transversely striated distribution pattern arranged throughout the fiber length in longitudinal cryosections (Fig. 1b-b’’). To localize the internal membrane domain in muscle fibers in which SVCT2 distributes, longitudinal cryosections of the LDA muscle were co-incubated with the anti-SVCT2 antibody and monoclonal antibodies against the structural proteins titin, desmin, slow myosin heavy chain (MHC), ryanodine receptor (RyR), slow C-protein, and myomesin. We found no co-localization of SVCT2 with the sarcomeric proteins titin (I-band) (Fig. 1c-c’’) and desmin (Z-disc) (Fig. 1d-d’’). Interestingly, SVCT2 showed a partial co-localization with the A-band marker slow MHC (Fig. 1e-e’’), and with the SR marker RyR (Fig. 1f-f’’). SVCT2 exhibits a high degree of co-distribution with slow C-protein at the A-band level (Fig. 1g-g’’) and a high degree of co-localization with the structural protein myomesin (M-band) (Fig. 1h-h’’), specifically at the center of the sarcomere. To further assess the co-localization of SVCT2 with slow C protein and myomesin, discrete regions of interest (ROIs) at 5 sarcomeric regions (Z-disc and A-band in each side, plus M-band) from longitudinal cryosections of slow-twitch fibers were subjected to topological fluorescence intensity analyses. The fluorescence intensity of each ROI was separated into their respective channels and plotted (Figs. 1g’’’ and h’’’). The results show that the intensity of the signals of SVCT2 compared with slow C protein display a partial overlap, with a central peak of fluorescence for SVCT2 flanked by two slow C protein peaks within the A-band (Fig. 1g’’’). The intensity and distribution of the signals between SVCT2 and myomesin are similar and display a peak at the M-band level, reinforcing the idea that these proteins exhibit a high degree of co-localization (Fig. 1h’’’). To complement these results, and considering our previous findings showing that SVCT2 is expressed in human oxidative muscle fibers [26], we aimed to evaluate the intracellular distribution of the transporter in longitudinal cryosections of human lumbar slow-twitch muscles. As in avian muscles, SVCT2 exhibits a high degree of co-distribution with myomesin in human skeletal muscles (Figs. 2a-a’’, b-b’’), evidenced by the close overlap of their spatial labeling (Fig. 2b’’) and by quantification of their fluorescence intensity in different sarcomeric regions (Fig. 2c). Our findings show that the intracellular distribution of SVCT2 in slow muscle fibers is conserved between species. Also, considering the regular and highly conserved structure of the sarcomere and associated organelles, our results suggest that SVCT2 localizes in internal membrane regions exhibiting topographical proximity to the M-band.

**Fig. 3:**
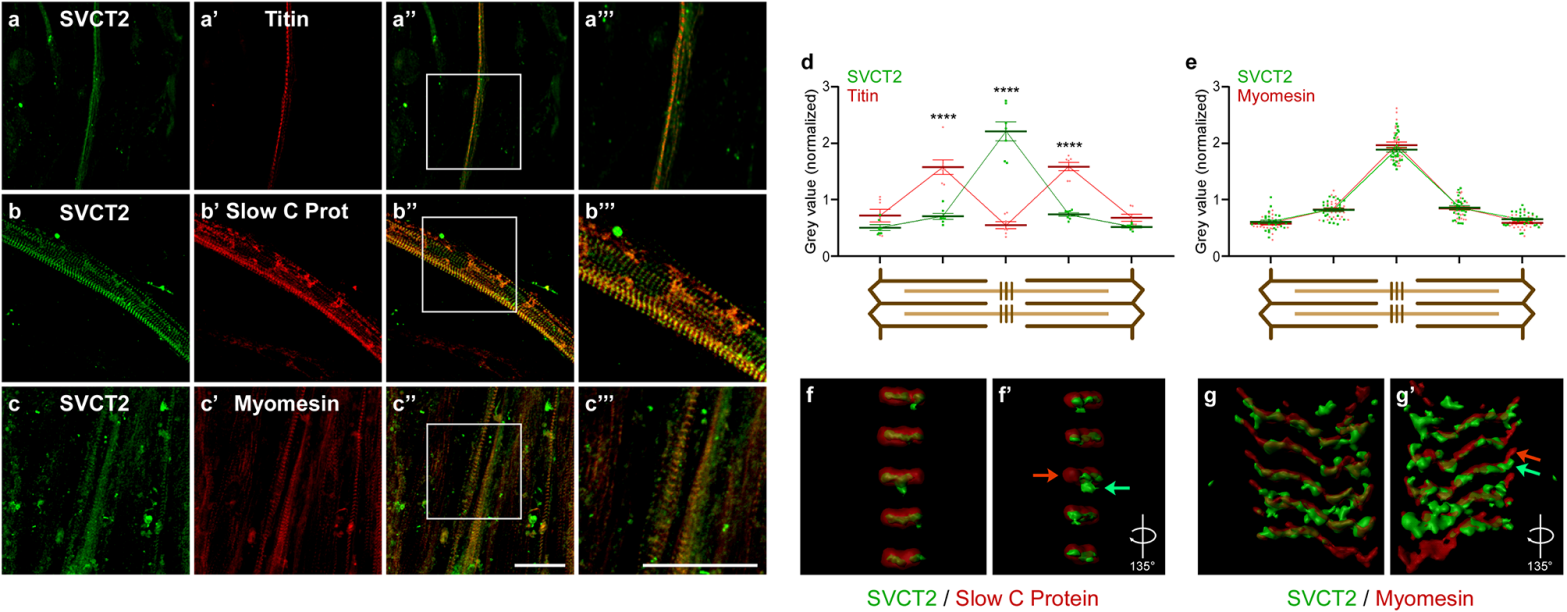
SVCT2 distributes in a striated intracellular pattern in primary cultures of chicken myotubes. (**a-c’’’**) Primary cultures of chicken myotubes were differentiated for 4 days and co-stained with anti-SVCT2 (green) along with antibodies against titin (**a-a’’’**), slow C protein (**b-b’’’**), or myomesin (**c-c’’’**). Magnified images from the squared regions show colocalization. Scale bar = 30 μm. (**d-e**) Quantification of fluorescence intensities throughout the sarcomeres (schemes below plots) evidence the differential distribution between SVCT2 and titin (**d**), as well as the close codistribution of SVCT2 and myomesin (**e**) at the M-band level. Plots represent the mean ± SEM of 7 (titin) and > 20 sarcomeres (myomesin; dots) from 3 different immunohistochemical staining. ****p < 0.0001, Anova. (**f-g’**) A z-stack of images obtained through confocal microscopy was processed to obtain the 3D representation of SVCT2 and slow C protein (**f-f’**) or SVCT2 and myomesin (g-g’) distribution. Straight (**f**,** g**) and rotated (**f’**,** g’**) images show that the distribution of SVCT2 at the M-band level occupies a different localization in the z plane than sarcomeric proteins

As SVCT2 is expressed in cultured myotubes obtained from chicken myoblasts [31], we next evaluated the subcellular localization of SVCT2 in in vitro cultured myotubes obtained from chicken embryo myoblasts (stage HH37) differentiated for 4 days. For immunohistochemistry assays, chicken myotubes were fixed and subsequently incubated with antibodies raised against SVCT2, along with antibodies for titin, slow C-protein, and myomesin. As seen in Fig. 3, cultured myotubes display a discrete sarcomerization, where SVCT2 exhibits a transverse striatal distribution pattern that is excluded from titin localization (I-band) (Fig. 3a-a’’’), partially colocalizes with the A-band marker slow C-protein (Fig. 3b-b’’’) and displays maximum colocalization with the M-band protein myomesin (Fig. 3c-c’’’), as was observed in vivo. Quantification of fluorescence intensity in different sarcomeric regions shows a complete exclusion of the labeling between SVCT2 and titin (Fig. 3d), while the fluorescence peaks of SVCT2 and myomesin co-localize at the M-band level (Fig. 3e). For a deeper analysis of the intracellular distribution of SVCT2, z-stacks of images obtained through confocal microscopy from immunocytochemical staining were reconstructed to obtain the 3D representation of SVCT2 and slow C protein (Fig. 3f-f’) or SVCT2 and myomesin (Fig. 3g-g’) distribution (see Supplementary videos 1 and 2). Straight *en face* representation confirms that SVCT2 distributes in a vesicle-like pattern at the M-band level (Fig. 3f, g); interestingly, a 135° rotation (Figs. 3f’, g’) of the images reveals that SVCT2 occupies a different localization in the z plane than sarcomeric proteins, as discrete regions display staining for only one labeling (see arrows in Figs. 3f’, g’ and supplementary videos 1 and 2).

**Fig. 4:**
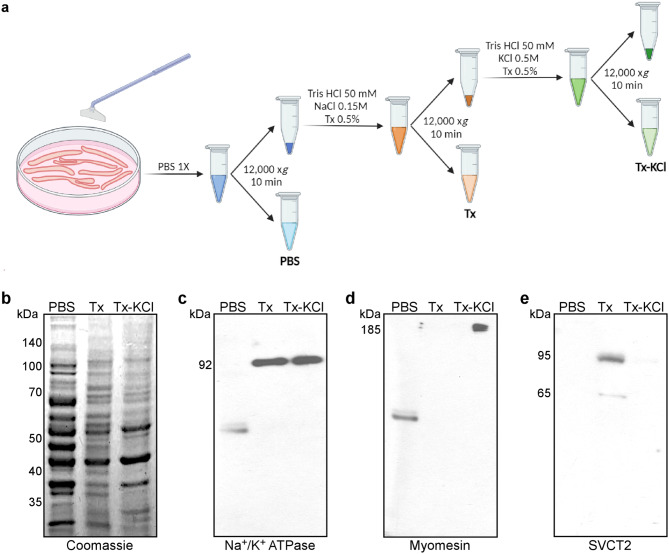
SVCT2 distributes in a membrane-enriched fraction in primary cultures of chicken myotubes. (**a**) Flow chart. Protein extracts enriched in cytosolic proteins (PBS), in membrane proteins (Tx), as well as in extracellular matrix and nuclear proteins (Tx-KCl) were obtained from chicken myotubes at day 4 of differentiation. (**b**) All steps of fractionation were separated by PAGE and stained with Coomassie Blue. (**c-e**) Western blots were performed using antibodies directed against Na^+^/K^+^ ATPase (**d**), myomesin (**e**), and SVCT2 (**f**) proteins

As a first hint to evaluate the segregation of SVCT2, cytosolic and membrane proteins were sequentially extracted from differentiated chicken myotubes in PBS followed by a detergent-containing (Tx) buffer, respectively. Insoluble proteins where further extracted in a buffer containing detergent plus high ionic strength (0.5 mM KCl; Tx-KCl) (Fig. 4a). Protein extracts were separated by PAGE-SDS (Fig. 4b) and subjected to Western blot analyses using antibodies for Na^+^/K^+^ ATPase, myomesin, and SVCT2 (Figs. 4c-e). As expected for a membrane-anchored protein, the Na^+^/K^+^ ATPase pump was detected in the Tx extract, while a remnant insoluble fraction was also found in the Tx-KCl soluble extract, as a 92 kDa-band (Fig. 4c). In turn, the structural protein myomesin was only detected in the Tx-KCl fraction as an immunoreactive-band of 185 kDa (Fig. 4d). SVCT2 was exclusively detected in the Tx extract, mostly as a 95 kDa-band and in a smaller proportion as a 65 kDa immunoreactive-band (Fig. 4e).

**Fig. 5:**
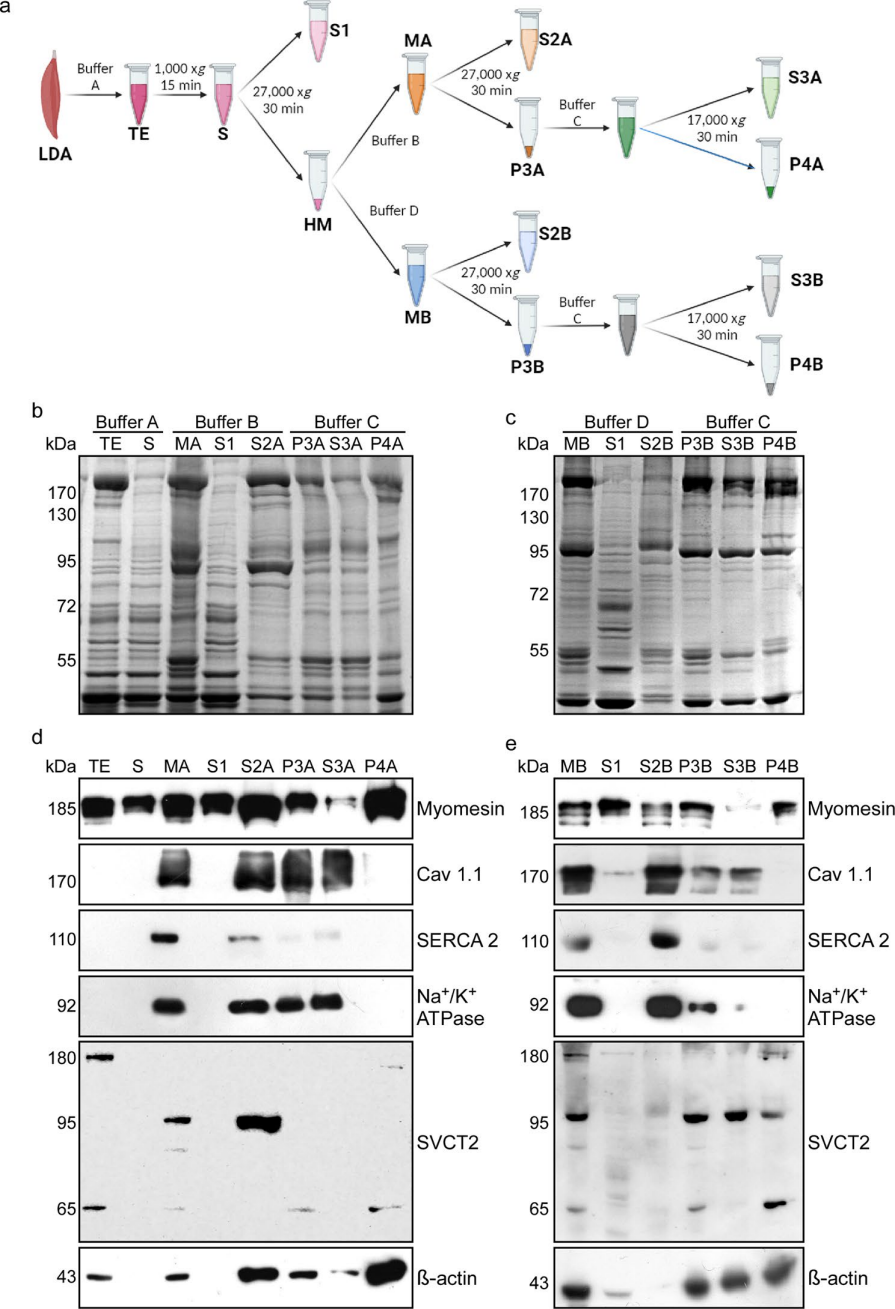
Subcellular ionic strength- and detergent-based fractionation reveals SVCT2 enrichment in a fraction containing SR proteins. **(a)** Flow chart. Protein extracts derived from adult chicken muscles were initially homogenized in buffer A (total extract, TE) and centrifuged. The resulting supernatant (S) underwent a subsequent centrifugation step, leading to the separation of a pellet containing heavy microsomes and a supernatant (S1). The heavy microsome fraction was further resuspended in either buffer B (MA lane) or buffer D (MB lane) followed by centrifugation, resulting in two distinct supernatants (S2A and S2B, respectively) and pellets that were separately resuspended in buffer C (P3A and P3B, respectively). Lastly, P3A and P3B were centrifuged, resulting in two supernatants (S3A and S3B) and two final pellets (P4A and P4B). (**b**,** c**) Representative electrophoretic migration of skeletal muscle protein fractions obtained through ionic strength and detergent treatment analyzed in 10% w/v acrylamide gel stained with Coomassie Blue. (**d**,** e**) Representative Western blot analyses using primary antibodies against myomesin, Cav1.1, SERCA2, Na^+^/K^+^ ATPase, SVCT2, and β-actin

Based on the structural conformation of SVCT2 as a 12-transmembrane domains protein, its segregation in a detergent-soluble fraction in vitro, and its close distribution with the RyR in vivo, we reasoned that SVCT2 distributes in an internal membrane subdomain associated with the SR. It has been shown that sequential fractionation of skeletal muscle homogenates [45], combined with buffers having high ionic strength allows the dissociation of internal membrane domains as well as actin-myosin protein interaction [46–48]. Therefore, dissected slow-twitch chicken muscles were homogenized in a buffer A (25 mM Tris HCl pH 7.4; 100 mM NaCl; 1 mM EDTA; 1 mM EGTA, and 1 mM PMSF) to obtain a total extract (TE) and then subjected to low-speed centrifugation to separate myonuclei from a supernatant (S). The supernatant was then centrifuged at high-speed to obtain a second supernatant (S1) enriched in cytosolic (sarcoplasmic) proteins and a pellet of heavy microsomes (HM), sealed membrane-enriched structures formed after centrifugation that contain membrane-associated proteins (Fig. 5). Considering the physicochemical properties that determine the association of proteins to biological membranes, the HM fraction of slow muscle proteins was subjected to two sub-fractionation procedures. In the first protocol, increasing concentrations of NaCl were applied to dissociate possible interactions between the different membrane-associated proteins with structural proteins of the sarcomere, followed by the non-ionic detergent Triton X-100 to solubilize the membrane-associated proteins in the HM fraction (ionic strength-detergent fractionation). Thus, the HM was resuspended in a buffer B (MA) having high NaCl concentration (500 mM) and again centrifuged at high speed, to obtain a supernatant (S2A) and a pellet (P3A). Finally, the P3A pellet was resuspended in a buffer C containing 500 mM NaCl plus 0.5% Triton X-100 to further obtain a supernatant (S3A) and a pellet (P4A) containing highly insoluble proteins (Fig. 5a). All steps of fractionation were separated by PAGE and subsequently stained with Coomassie Blue (Figs. 5b, c). An evident enrichment of proteins whose molecular weight ranged from 43 to 55 kDa, 95 kDa, and higher than 170 kDa was observed in a 30 µg aliquot of a total protein extract, used here as loading control (Fig. 5b). To determine the localization of SVCT2, Western blot assays were performed on the different fractions and compared with the immunodetection with antibodies against the structural protein myomesin as well as for several proteins associated with different internal membranes. Interestingly, Western blot analyses showed that the anti SVCT2 antibody recognized three-bands of 180, 95, and 65 kDa in the crude extract (data not shown). From these, the 180 and 65 kDa-bands were solubilized in the total extract, whereas the 95 kDa-band was the main polypeptide incorporated into the MA fraction (Fig. 5d). Our results show that the 95 kDa SVCT2-positive-band of the HM is also enriched in fraction S2A. On the other hand, membrane proteins such as the Cav1.1 receptor and the Na^+^/K^+^ ATPase are mostly concentrated in the MA, S2A, P3A, and S3A fractions as unique immunoreactive-bands of 170 and 92 kDa, respectively. Remarkably, the distribution of SVCT2 closely resemble the solubilization of the 110 kDa-band immunodetected with the antibodies raised against SERCA 2, a specific Ca^**2**+^-ATPase of the SR membrane. In turn, the P4A fraction contains the structural proteins myomesin and β-actin, as indicated by their immunoreactive-bands of 185 and 43 kDa, respectively, confirming that this fraction contains mainly insoluble cytoskeleton-associated proteins (Fig. 5d). In the second sub-fractionation protocol, the HM fraction was initially resuspended in a buffer D (MB) containing 100 mM NaCl and 0.5% Triton X-100 to obtain S2B and P3B; finally, the P3B pellet was resuspended in a buffer C containing 500 mM NaCl buffer plus 0.5% Triton X-100 (detergent-ionic strength fractionation). Also, fractions were separated in a PAGE-SDS gel and stained with Coomassie Blue (Fig. 5c). Western blot analyses showed that the membrane proteins Cav1.1, SERCA 2, and the Na^+^/K^+^ ATPase were mainly localized in the MB and S2B fractions. Structural proteins such as myomesin (185 kDa) and β-actin (43 kDa) mostly localized in the insoluble P4B fraction. However, in contrast to what was observed in the previous fractionation, SVCT2 (95 kDa) was mainly detected in the P3B and S3B fractions (Fig. 5e). Altogether, our detergent-based fractionation data reveal that SVCT2 distributes in a membrane organelle. As high ionic strength is required to fully solubilize SVCT2 from the HM fraction, it is likely that SVCT2 is also closely associated to structural proteins.

**Fig. 6:**
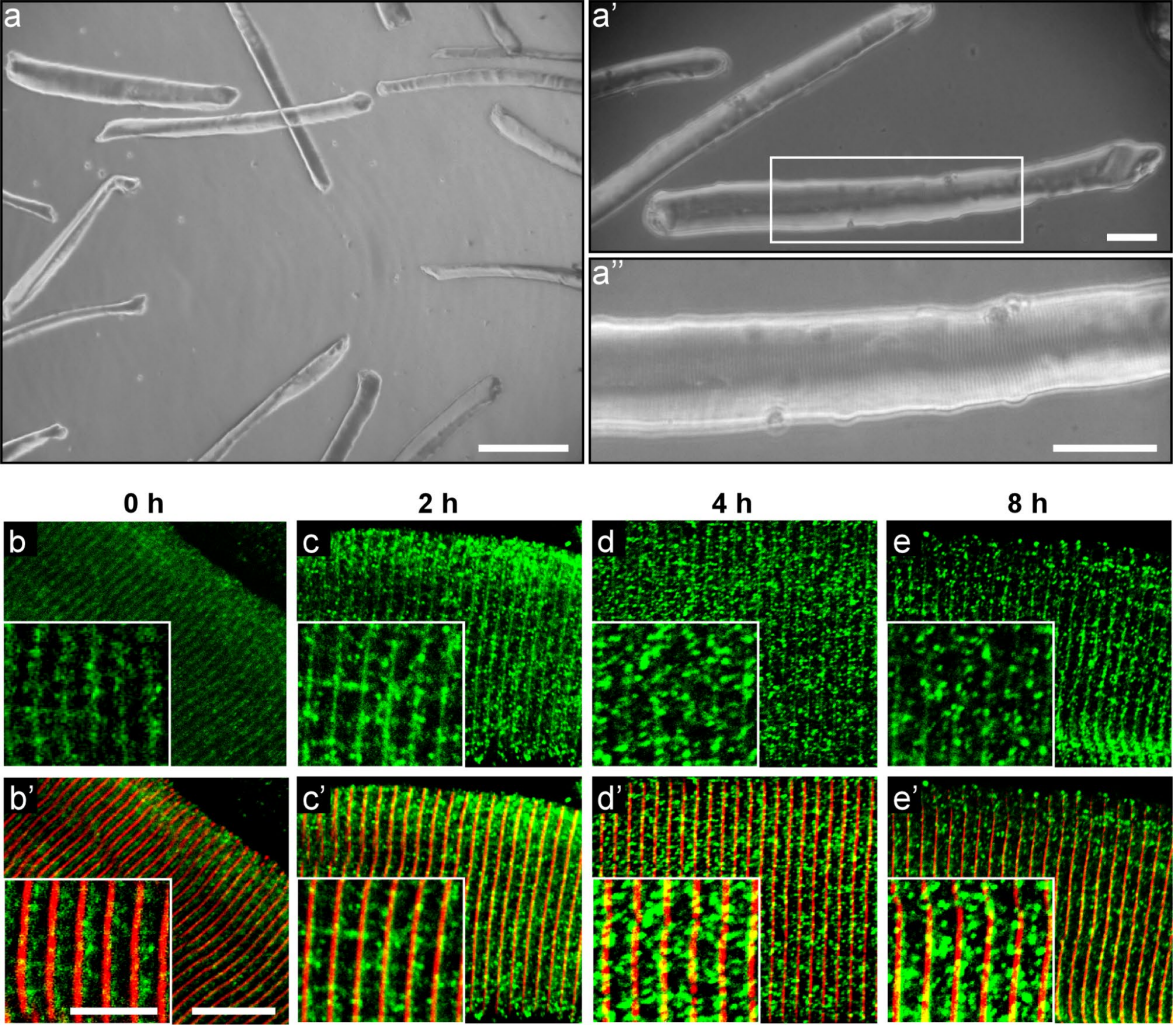
SVCT2 alters its intracellular distribution in skeletal muscle fibers in response to electrical stimulation at 20 Hz. (**a-a’’**) Phase-contrast images of cultured skeletal muscle fibers isolated from the FDB muscle of adult mice and magnifications. Scale bar = 200 μm (**a**) and 50 μm (**a’**,** a’’**). (**b-e’**) Representative images of isolated fibers from adult mouse FDB muscles electrically stimulated with 270 pulses at low frequency of 20 Hz at 0 (**b-b’**), 2 (**c-c’**), 4 (**d-d’**), and 8 h (**e-e’**). The fibers were fixed and immunocytochemistry to SVCT2 (green) and myomesin (red) were performed. Scale bar = 10 μm and 5 μm (insets)

We have previously demonstrated that the expression of SVCT2 is increased in response to membrane depolarization of chicken cultured myotubes with KCl [31]. Interestingly, it is well known that muscle activity induces the translocation of the glucose transporter GLUT4 from an intracellular compartment to the sarcolemma [49, 50]. Based on this evidence, we next aimed to analyze the distribution of SVCT2 in response to electrical stimulation. With this aim, we isolated myofibers from the FDB muscle (Fig. 6a-a’’) and stained them to reveal myomesin and SVCT2. We observed that a high proportion (~ 50%) of fibers were positive for SVCT2 staining (Fig. 6b-b’), a feature likely related to the oxidative nature, not only of type I, but also of type IIa fibers, which are abundant in this muscle [51]. Next, isolated fibers were subjected to low-frequency stimulation (20 Hz) (Figs. 6b-e and b’-e’) to mimic the activity of motor neurons innervating slow skeletal muscles [52]. Indeed, this electrical stimulation pattern efficiently increases the levels of slow fiber transcripts [53]. As expected, immunohistochemistry assays showed that the distribution of the structural protein myomesin was not affected by membrane depolarization. In turn, the immunoreactivity for SVCT2 becomes progressively lost from its transversely striated distribution and becomes concentrated in a vesicle-like pattern throughout the sarcoplasm after 2, 4, and 8 h of stimulation (Fig. 6b-e and b’-e’). These results suggest that SVCT2 could distribute dynamically in different intracellular compartments as a response to functional requirements.

## Discussion

According to the demanding nature of motor performance, skeletal muscles engage in continuous contractile activity, leading to varying levels of oxidant species production. The ensuing antioxidant responses orchestrated by molecular players must be intricately coordinated in the subcellular space to effectively prevent the increase of ROS. In this work, we show that SVCT2 has a conserved pattern of expression within slow muscle fibers and our analysis suggest that it is anchored to a portion of the SR mainly associated with the M-band of the sarcomere and redistributes according to functional requirements after skeletal muscle fiber electrical stimulation at low frequency.

In muscles, the antioxidant mechanisms are compartmentalized either in the sarcoplasm or associated with the membranous components, mainly mitochondria and the endoplasmic reticulum [9, 54]. Mitochondria contain several antioxidant defences including superoxide dismutase enzymes (SOD); while SOD2 is located in the mitochondrial matrix, SOD1 is distributed in the intermembrane space [55]. Mitochondria, peroxisomes, and the endoplasmic reticulum also host several antioxidant enzymes [54, 56–60]. In this context, the main pool of ROS in strenuous oxidative or high-intensity exercise is counteracted by NADPH oxidases (NOX proteins), with NOX2 and NOX4 located in the sarcolemma, transverse tubules, and the SR of muscle cells [9, 61–63]. In line with our present results, NOX activity is significantly higher in the slow-twitch oxidative soleus muscle compared with the fast twitch glycolytic gastrocnemius [63, 64]. Interestingly, electrical stimulation leading to the expression of slow-type muscle genes (as the one used in this work) induces an increase in ROS production by NOX2, possibly being part of the necessary changes for fiber-type transition from fast to slow phenotype [65].

During muscle activity, NOX-derived ROS could modulate signaling pathways such as NF-κB or the cytosolic concentration of Ca^2+^ that, in turn, upregulates the serine/threonine phosphatase calcineurin, which selectively stimulates slow fiber-specific gene promoters [66]. In this context, strong evidence suggests the potential influence of vitamin C in the epigenomic activity by regulating DNA demethylation in different processes including embryonic development, cellular reprogramming, cancer, and peripheral neuropathy, among others [67, 68]. In this regard, findings in other cell types reveal that ascorbate uptake through SVCT2 in skeletal muscle fibers could control ROS accumulation not only as part of an exogenous non-enzymatic antioxidant mechanism but also by modulating signaling pathways in different cell compartments. For instance, in Schwann cells, the uptake of ascorbic acid through SVCT2 promotes myelination of peripheral axons by increasing DNA demethylation, transcription of pro-myelinating genes, and stabilizing collagen helices to form the basal lamina for myelin formation [68–72]. Accordingly, ascorbic acid is a cofactor for endoplasmic reticulum-localized collagen hydroxylases [13]. In addition, a short isoform of SVCT2 has been identified in the endoplasmic reticulum of glioblastoma cells, where it has been associated to collagen synthesis and invasiveness [73]. Also, in human embryonic kidney-derived HEK293 cells, the uptake of ascorbic acid promotes an active-dependent trafficking of SVCT2 via intracellular early secretory and endocytic-recycling compartments to communicate the plasma membrane with the endoplasmic reticulum and mitochondria [34, 38]. Interestingly, in these cellular models, vitamin C treatment triggered the mobility of SVCT2 towards the plasma membrane via the endocytic and the secretory pathways as acute and post-acute responses, respectively [38]. Supporting this idea, the localization of SVCT2 throughout the secretory and endosomal routes is affected in a cellular model of Huntington’s disease [74]. These findings described a trafficking mechanism that involves SVCT2 trapping in the endoplasmic reticulum and mobilization to the plasma membrane according to substrate availability [34, 37, 38, 75, 76].

A major finding of the present work is the subcellular localization of SVCT2 in a membrane compartment distributed at the level of the central sarcomeric M-band. Interestingly, besides its role as the anchoring point of thick myosin filaments and other crucial structural sarcomeric proteins, the M-band also concentrates proteins involved in cell signaling as well as those involved in energy production and consumption. For instance, a proportion of glycolytic enzymes, such as creatine kinase [77] and β-enolase [78], have been shown to bind the M-band, suggesting that this sarcomeric domain could serve crucial roles during periods of heightened muscle activity [78], or muscular damage, such as in myocardial infarction [79]. In the context of our present findings, it is particularly interesting that the cAMP-dependent protein kinase A (PKA) and its effectors are concentrated at the M-band and contribute to its organization [80]. PKA signaling is enriched in slow compared to fast skeletal muscles, suggesting that its expression is regulated by different activity patterns [81]. PKA also regulates specific and reversible gene expression programs in skeletal muscle [82–85]. Indeed, the PKA-dependent phosphorylation of SVCT2 in osteoblasts is required for the translocation of intracellular SVCT2 to the plasma membrane [39]. Thus, our current findings open the possibility that a similar compartmentalization mechanism of phosphorylation-dependent translocation of SVCT2 could occur at the M-band level of slow muscle fibers (see below).

Cumulative evidence has demonstrated that M-band proteins interact with membranous structures. For instance, obscurin, a giant integral M-band protein, contains kinase and RhoGEF domains that associate with membrane phospholipids and other signaling proteins [86–88] and has been shown to play a major role in the organization of the sarcolemmal and SR membrane architecture [80]. Our results suggest that SVCT2 is distributed in the SR, the organelle that concentrates calcium for muscle contraction. The SR is a heterogeneous membrane organelle encompassing SR vesicles, the longitudinal SR segment that distributes around the central region of the sarcomere (where the M-band is located), and, to a lesser extent, fragments associated to sarcolemmal T-tubules [89]. Our immunohistrochemistry data reveal that SVCT2 displays a partial co-distribution with the A-band enriched slow C-protein, the slow MHC, and the SR protein RyR, as well as a strong co-distribution with the M-band protein myomesin. Accordingly, our subcellular protein fractionation experiments utilizing a sucrose gradient revealed that SVCT2 is solubilized in the HM fraction, enriched in longitudinal portions of the intact SR, as also demonstrated by the co-solubilization with SERCA2. However, although a detergent treatment of HMs solubilized SERCA2, membrane solubilization did not yield SVCT2 extraction, which was only accomplished after a high ionic strength treatment of the microsomal fraction. In this regard, it is relevant to point out that the sarcomeric proteins myomesin, and particularly β-actin, follow a similar solubilization pattern than SVCT2. Together, these findings further support the idea that SVCT2 is associated to an SR membrane domain closely attached to structural M-band proteins, such as obscurin.

Finally, our findings also reveal that upon electrical stimulation of isolated skeletal muscle fibers, SVCT2 modifies its continuous distribution typically associated with the M-band and becomes dispersed across a broader area, in a vesicle-like pattern. In this regard, it has been shown that SVCT2 traffics from intracellular compartments to the plasma membrane after stimulation with ascorbic acid [37, 38] and prostaglandin E2 [39]. Also, the trafficking of SVCT2 among different cellular compartments is associated to specific cell requirements. For instance, adaptative up-regulation of SVCT2 has been described as a response of increased ROS levels in senescent human fibroblasts, where it translocates from an intracellular distribution to the plasma membrane [90]. Also, the exclusive expression of SVCT2 in the mitochondria of breast cancer cells has been associated to cell survival in the context of pro-oxidant environments [91].

We envisage that a crucial future development in the field is to determine the functional implication of SVCT2 distribution in the central longitudinal portion of the SR. In this regard, the longitudinal SR actively participates in calcium removal from the cytosol, an activity operated by the SERCA2 that pumps calcium from the cytoplasm to the lumen of the SR to allow a new contraction event [92, 93]. In this context, it is also relevant mentioning recent evidence showing that repetitive activation of muscle contraction resulting in calcium depletion from intracellular stores induces extracellular calcium entry [94]. This mechanism relies on a strong remodelling of T-tubules, as they extend longitudinal tubes that run parallel and make physical contacts with flat cisternal stacks of the longitudinal SR to form new sites of interaction termed calcium entry units [95]. In the context of the excitation/contraction coupling between the SR and the T-tubules that takes place in the triad, the redox state of RyR channels is a crucial determinant of the channel activity, as oxidation of the highly susceptible SH groups of the receptor induces calcium release from SR vesicles, activates membrane-associated RyR channels, and modifies ryanodine binding to SR membranes [96]. Based on this evidence, we speculate that the dynamic distribution of SVCT2 from the central region of the sarcomere, possibly anchored to M-band proteins, towards new intracellular locations after repetitive stimulation could be a crucial step to uptake ascorbic acid as a mechanism to control the redox status and modulate calcium influx from the cytoplasm or from the extracellular space to the SR. Most certainly, characterizing the novel SVCT2 distribution elicited by muscle fiber excitation represents a crucial step to improve our understanding of striated muscle physiology and function. Future experiments could shed light on the precise dynamic localization of SVCT2 at the SR level, and on the potential function of this intracellular transporter to modulate redox levels; likewise, such mechanism could help calcium availability and/or muscle signaling, particularly in response to stressors like exercise.

## Electronic supplementary material

Below is the link to the electronic supplementary material.

Supplementary Material 1

Supplementary Material 2

## Data Availability

The datasets during and/or analysed during the current study available from the corresponding author on reasonable request.
